# Binding site residues control inhibitor selectivity in the human norepinephrine transporter but not in the human dopamine transporter

**DOI:** 10.1038/srep15650

**Published:** 2015-10-27

**Authors:** Jacob Andersen, Kristoffer B. Ringsted, Benny Bang-Andersen, Kristian Strømgaard, Anders S. Kristensen

**Affiliations:** 1 Department of Drug Design and Pharmacology, University of Copenhagen, Jagtvej 162, DK-2100 Copenhagen, Denmark; 2 Lundbeck Research Denmark, H. Lundbeck A/S, Ottiliavej 9, DK-2500 Valby, Denmark

## Abstract

The transporters for norepinephrine and dopamine (NET and DAT, respectively) constitute the molecular targets for recreational drugs and therapeutics used in the treatment of psychiatric disorders. Despite a strikingly similar amino acid sequence and predicted topology between these transporters, some inhibitors display a high degree of selectivity between NET and DAT. Here, a systematic mutational analysis of non-conserved residues within the extracellular entry pathway and the high affinity binding site in NET and DAT was performed to examine their role for selective inhibitor recognition. Changing the six diverging residues in the central binding site of NET to the complementary residues in DAT transferred a DAT-like pharmacology to NET, showing that non-conserved binding site residues in NET are critical determinants for inhibitor selectivity. In contrast, changing the equivalent residues in the central site of DAT to the corresponding residues in NET had modest effects on the same inhibitors, suggesting that non-conserved binding site residues in DAT play a minor role for selective inhibitor recognition. Our data points towards distinct structural determinants governing inhibitor selectivity in NET and DAT, and provide important new insight into the molecular basis for NET/DAT selectivity of therapeutic and recreational drugs.

Transporters for the biogenic monoamine neurotransmitters norepinephrine, dopamine and serotonin (NET, DAT and SERT, respectively) are integral membrane proteins that regulate monoaminergic signalling in the brain by performing sodium- and chloride-coupled uptake of neurotransmitters from the extracellular space into neurons^1^. Inhibitors of the three monoamine transporters (MATs) increase the extracellular concentration of monoamines, and are widely used in the treatment of psychiatric diseases and as illicit psychostimulant drugs^2^. The selectivity profile of MAT inhibitors across NET, DAT and SERT is critical for their therapeutic profile and/or abuse potential. Specifically, antidepressant medications, including the selective serotonin reuptake inhibitors and tricyclic antidepressants (TCAs), predominantly block SERT and/or NET with little or no affinity for DAT^3^, whereas psychostimulants, like cocaine and amphetamines, target all three MATs, albeit their reinforcing properties and abuse potential are attributed to blockade of DAT^4^,^5^. Interestingly, some compounds show potent inhibition of DAT but no cocaine-like behaviour in animal models^6^,^7^,^8^. This is not fully understood but may be explained by a concomitant activity at sigma-receptors, slow binding rate to DAT or conformational selectivity (i.e. bias for binding to a distinct conformation of DAT compared to cocaine)^9^. The lack of stimulant activity could potentially be exploited in the development of treatments of stimulant abuse, and several DAT inhibitors have been pursued as pharmacotherapies for cocaine addiction^9^.

Current structural understanding of human MATs is based on x-ray crystal structures of bacterial and invertebrate homologs, which include the bacterial amino acid transporters LeuT and MhsT and the *Drosophila melanogaster* DAT (dDAT)^10^,^11^,^12^,^13^. These structures have established that MATs share a conserved topology consisting of 12 transmembrane domains (TMs) arranged in a barrel-like bundle with the substrate binding site (denoted the S1 site) located in the core of the protein structure (Fig. 1). Although x-ray crystal structures of LeuT in complex with antidepressant drugs have suggested that some MAT inhibitors potentially bind in a vestibular site (denoted the S2 site) in the extracellular permeation pathway^14^,^15^,^16^, recent x-ray crystal structures of dDAT have shown that the binding site for several classical MAT inhibitors overlaps the central S1 site (Fig. 1)^13^,^17^,^18^. Together with mutational^19^,^20^,^21^,^22^,^23^, biochemical^24^,^25^,^26^,^27^, and computational^24^,^28^,^29^,^30^,^31^,^32^,^33^,^34^ studies of inhibitor binding in MATs, these structures provide compelling evidence that the high affinity binding site for most, if not all, MAT inhibitors overlaps the central S1 site. In contrast, the S2 site has been suggested to harbour an allosteric inhibitor site in human MATs^35^.

Resolving the molecular differences among NET, DAT and SERT that control selective inhibitor binding is important for structure-based design of MAT inhibitors with fine-tuned selectivity profiles. Within the S1 site, non-conserved residues can confer important differences among MATs in the shape and physicochemical properties of the inhibitor binding pocket, and importantly change the number and strength of direct interactions with the bound inhibitor molecule. The central location of the S1 site means that inhibitors must traverse from the extracellular space through a funnel-like extracellular entrance pathway, that include the S2 site, to reach the S1 site (Fig. 1). Thus, non-conserved residues in the extracellular entrance pathway, including the extracellular loop 4 (EL4) that is an important gatekeeper for inhibitor access to the entrance pathway^10^,^36^,^37^, may confer differential ability among MATs for transient interactions with the inhibitor as it travels through the pathway, hereby controlling diffusion rates to and from the S1 site. Taken together, non-conserved residues in the S1 site and in the extracellular entrance pathway, including the S2 site and EL4, are thus obvious candidates for controlling selective inhibitor binding in MATs. Previous studies have shown that it is possible to tweak ligand selectivity by switching non-conserved S1 residues between MATs and/or related neurotransmitter transporters^21^,^29^,^33^,^38^,^39^,^40^,^41^,^42^. This implies that inhibitor selectivity is controlled by S1 residues in MATs, and is in accordance with the general idea that ligand affinity is mainly determined by specific ligand/protein interactions in the primary binding pocket^43^. In contrast, it was recently demonstrated that some inhibitors have different rates of binding (association) to DAT and SERT whereas their rates of unbinding (dissociation) were similar, indicating that diffusion of inhibitors through the entry pathway contribute to MAT inhibitor selectivity^44^. Notable, this is in analogy to G protein-coupled receptors, where binding of certain ligands is influenced *en route* to the binding site by residues in distal permeation sites close to the receptor surface^45^,^46^. In the context of our current understanding of MAT structure, a complex picture is emerging for the mechanism underlying MAT inhibitor selectivity that suggests that selectivity for some inhibitors is conferred by differences among MATs in the extracellular entrance pathway, whereas differences within the S1 binding site determine selectivity for other inhibitors.

The human NET and DAT (hNET and hDAT, respectively) share a particular high homology with almost 80% overall identity in their amino acid sequence (Supporting Fig. 1), and both transporters can convey dopamine and norepinephrine across cellular membranes^47^. Despite the close relationship between hNET and hDAT, multiple NET- and DAT-selective inhibitors have been developed. It is noteworthy that several NET inhibitors have very high selectivity for NET over DAT (up to several orders of magnitude), while it has been more challenging to develop inhibitors with equivalent degree of selectivity for DAT over NET^48^,^49^. The molecular basis underlying inhibitor selectivity for NET and DAT is poorly understood, including the relative contribution of non-conserved residues in the S1 site and the extracellular entrance pathway. Early studies employing chimeric NET/DAT constructs identified two regions spanning TM 1–3 and TM 5–8, respectively, to contain important determinants for inhibitor selectivity^50^,^51^,^52^. The S1 site is formed by residues on TMs 1, 3, 6 and 8 and both regions thus encompass residues in the central S1 binding site. However, the TM 5–8 region also includes EL4, and it has previously been shown that introduction of a single DAT residue into EL4 of NET affect the potency of NET inhibitors, suggesting that residues in the extracellular permeation pathway may also play a role for inhibitor selectivity in NET and DAT^42^,^53^.

In the present study, we systematically examine the role of non-conserved residues in hNET and hDAT that are located within the S1 site and in the extracellular permeation pathway, including the S2 site and EL4, for selective inhibitor recognition. We find that non-conserved S1 residues in hNET are primary determinants for inhibitor selectivity. Surprisingly, the corresponding residues in hDAT do not hold an equivalent important role for inhibitor selectivity. This indicates that selective inhibition in these two closely related MATs is achieved by distinct mechanisms. Our findings provide new insight into the molecular basis for inhibitor selectivity between hNET and hDAT, which may be used in the rational design and development of novel therapeutic agents targeting hNET and hDAT with tailor-made selectivity profiles.

## Results

### Determinants for inhibitor selectivity in hNET and hDAT

**Figure 1:**
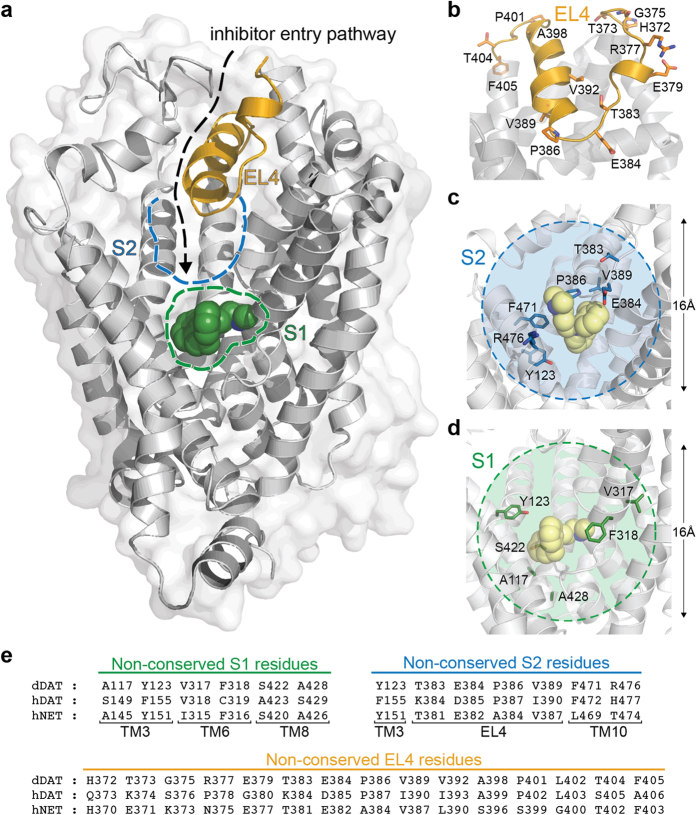
The extracellular entry pathway for inhibitors in hNET and hDAT. (**a**) The extracellular entry pathway for inhibitors is illustrated on the nortriptyline-bound dDAT x-ray crystal structure (PDB ID 4M48). Location of the S1 and S2 sites are indicated by green and blue dashed lines, respectively, and the EL4 region is shown in yellow. Nortriptyline is shown as green spheres. (**b**) Close-up view of the EL4 region in dDAT. The 15 non-conserved hNET/hDAT residues in EL4 are shown as sticks (dDAT numbering). (**c**) Close-up view of the S2 site in dDAT. Imipramine is shown as yellow spheres in the site equivalent to the imipramine binding site found in LeuT (PDB ID 2Q72). The seven non-conserved hNET/hDAT residues within 8Å of the S2 site are shown as blue sticks (dDAT numbering). (**d**) Close-up view of the S1 site in dDAT. Nortriptyline is shown as yellow spheres. The six non-conserved hNET/hDAT residues within 8 Å of the S1 site are shown as green sticks (dDAT numbering). (**e**) Amino acid sequence alignment between dDAT, hDAT and hNET showing the non-conserved hNET/hDAT residues within 8 Å of the S1 and S2 sites and the EL4 region. A complete amino acid sequence alignment between dDAT, hDAT and hNET is included in Supporting Figure S1.

**Figure 2:**
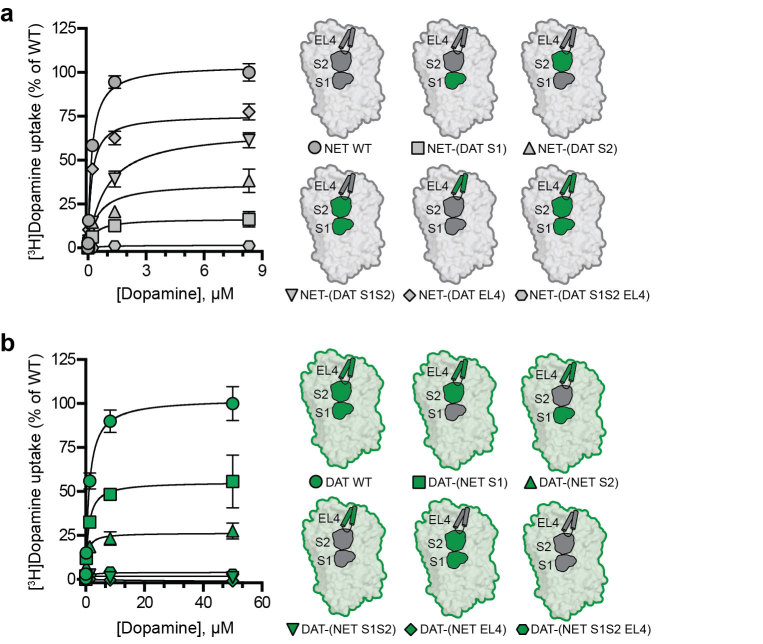
Uptake kinetics for hNET and hDAT mutants. [^3^H]dopamine uptake in COS-7 cells expressing hNET WT or hNET mutants (**a**) or hDAT WT or hDAT mutants (**b**). WT and mutant transporters were assayed in parallel, and the uptake of the mutants was normalized to the uptake of WT transporters. Data points represent mean ± s.e.m. from representative experiments carried out in triplicate. Schematic illustrations of the hNET (**a**) and hDAT (**b**) mutants are shown on the right. hNET mutants are shown on a grey background and regions inserted from hDAT are shown in green. hDAT mutants are shown on a green background and regions inserted from hNET are shown in grey. The uptake saturation curves shown are transformations of the [^3^H]dopamine competition uptake assays that were used determined dopamine *K*_M_ values. *K*_M_ and V_max_ values for dopamine uptake in addition to the specific point-mutations that are included in the hNET and hDAT mutants are summarized in Supporting Table S1.

In previous studies, we examined the role of non-conserved residues within the S1 site of human SERT (hSERT) and hNET for inhibitor selectivity by mutating all non-conserved residues to the corresponding residues in the other transporter, and thereby in principle transferred the S1 site from hNET into hSERT and vice versa. Hereby, we demonstrated that non-conserved S1 residues are key determinants for the hSERT/hNET selectivity of the two prototypical SERT selective inhibitors, escitalopram and fluoxetine^29^,^38^. Encouraged by the results from these previous studies, we wanted to perform a similar systematic analysis of non-conserved residues in the S1 site and in the extracellular entrance pathway (including EL4 and the S2 site) in hNET and hDAT. We defined the S1 site as the nortriptyline binding site in dDAT^13^ and the S2 site was defined as the pocket in dDAT equivalent to the imipramine binding site in LeuT^14^ (Fig. 1 and Supporting Fig. S1). Within this definition, we identified 56 and 36 residues located within 8Å of S1 and S2, respectively, in dDAT. Among these, six residues within the S1 site and seven residues within the S2 site are non-conserved between hDAT and hNET (Fig. 1 and Supporting Fig. S1). A single non-conserved residue (Phe155 in hDAT; Tyr151 in hNET) is located in the S1/S2 interface and was therefore included as part of both sites to give a total of 12 non-conserved residues in the S1/S2 binding region. Furthermore, 15 out of 37 residues within the EL4 region are non-conserved between hNET and hDAT (Fig. 1 and Supporting Fig. S1). We mutated all non-conserved residues within S1, S2 and EL4, respectively, in hNET and hDAT to the identity of the corresponding residues in the other transporter, and thereby in principle transferred these three regions individually between the two transporters (Fig. 2). Similarly, by mutation of all non-conserved residues within two or three regions in the same construct, we simultaneously transferred S1 and S2 or S1, S2 and EL4 between hNET and hDAT (Fig. 2).

To determine the ability of the resulting hNET and hDAT mutants to express to cell membrane with intact transport function, the ten mutated transporter constructs were initially expressed along with wild-type (WT) hNET and hDAT in COS-7 cells and assessed for their ability to transport [^3^H]dopamine (Fig. 2, Supporting Table S1). Four of the five hNET mutants showed robust uptake activity, and only the NET-(DAT S1S2 EL4) mutant, in which S1, S2 and EL4 simultaneously were transferred from hDAT into hNET, was devoid of any measurable uptake activity (Fig. 2 and Supporting Table S1). For the five hDAT mutants, function was retained in the mutants in which S1 or S2 individually was transferred from hNET, whereas simultaneous insertion of the S1 and S2 sites and/or EL4 from hNET into hDAT abolished transport function (Fig. 2, Supporting Table S1). We also examined the ability of the hNET and hDAT mutants to bind the cocaine analogue [^125^I]β-CIT using membrane preparations from COS-7 cells expressing the ten mutants. All functional hNET and hDAT mutants were capable of binding [^125^I]β-CIT (Supporting Table S1). Furthermore, the non-functional hDAT mutant DAT-(NET S1S2) displayed [^125^I]β-CIT binding, whereas the remaining non-functional mutants did not bind [^125^I]β-CIT (Supporting Table 1). In summary, seven of the ten hNET and hDAT mutants transported [^3^H]dopamine and/or bound [^125^I]β-CIT and could thus be subjected to pharmacological characterization to examine the role of non-conserved residues in the S1 and S2 sites and in EL4 for inhibitor selectivity.

### Role of non-conserved residues for inhibitor selectivity in hNET

**Figure 3:**
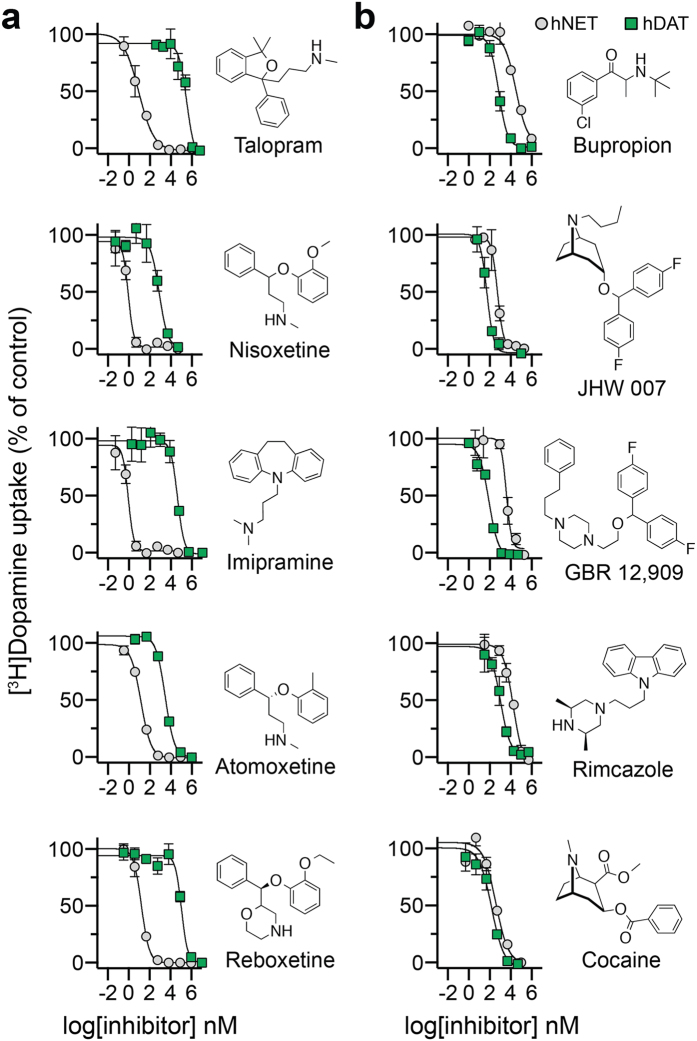
Inhibitor selectivity between hNET and hDAT. Inhibition of [^3^H]dopamine uptake by the five NET inhibitors (**a**) and the five DAT inhibitors (**b**) in COS-7 cells expressing hNET WT (grey circles) or hDAT WT (green squares). Data points represent mean ± s.e.m. from representative experiments carried out in triplicate. The inhibitory potency (*K*_i_) for the ten inhibitors are summarized in Supporting Table S2. The hDAT/hNET selectivity ratio was calculated as *K*_i_(hDAT WT)/*K*_i_(hNET WT) for the five NET inhibitors and were as follows: talopram, 17,531; nisoxetine, 412; imipramine, 1,555; atomoxetine, 160; reboxetine, 6,952. The hNET/hDAT selectivity ratio was calculated as *K*_i_(hNET WT)/*K*_i_(hDAT WT) for the five DAT inhibitors and were as follows: bupropion, 36; JHW 007, 6; GBR 12,909, 8; rimcazole, 16; cocaine, 2.

To examine the role of non-conserved residues within S1, S2 and EL4 for inhibitor selectivity, we selected five NET selective inhibitors and five DAT selective inhibitors (Fig. 3). This included inhibitors used in the treatment of depression (imipramine and atomoxetine) and attention-deficit hyperactivity disorder (atomoxetine), a smoking cessation agent (bupropion), a psychostimulant drug (cocaine), and pharmacological tool compounds with selectivity for NET (nisoxetine and talopram) or DAT (GBR 12,909, JHW 007 and rimcazole). hNET WT and hDAT WT were expressed in COS-7 cells, and the inhibitory potency of the ten compounds was determined in [^3^H]dopamine uptake inhibition assays (Fig. 3 and Supporting Table S2). In general agreement with previous findings^48^,^49^, inhibitor selectivity between hNET and hDAT were more pronounced for the five NET inhibitors (160- to 17,531-fold increased potency for hNET over hDAT) compared to the DAT inhibitors. Bupropion, JHW 007, GBR 12,909 and rimcazole had 6- to 36-fold higher potency towards hDAT compared to hNET while cocaine was equally potent on hDAT and hNET (Fig. 3). We also determined the binding affinity of the ten inhibitors at WT transporters by displacement of [^125^I]β-CIT binding from membrane preparations of COS-7 cells, and the binding affinities of the ten inhibitors were comparable with the inhibitory potencies determined in uptake inhibition assays (Supporting Table S3).

**Figure 4:**
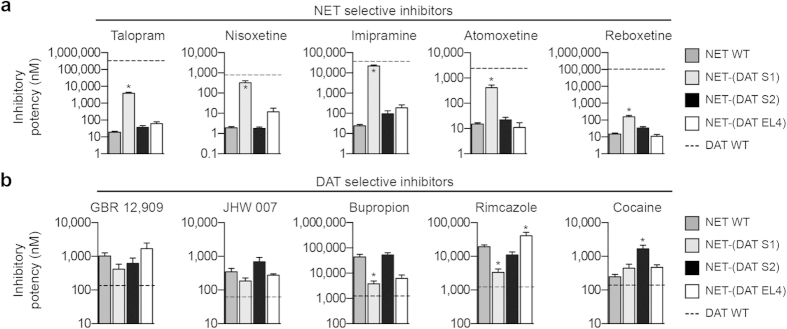
Pharmacological characterization of hNET mutants. The inhibitory potency of the five NET inhibitors (**a**) and the five DAT inhibitors (**b**) were determined in [^3^H]dopamine uptake inhibition assays at hNET WT, NET-(DAT S1), NET-(DAT S2), NET-(DAT EL4) and hDAT WT. The bars represent mean ± s.e.m. determined from 3–18 independent experiments each performed in triplicate. The inhibitory potency at hDAT WT is indicated by dashed line. Asteriks indicate that the inhibitory potency is significantly different compared to hNET WT (*p* < 0.05; one-way ANOVA with Dunnett’s multiple comparisons test). The *K*_i_ values for the ten inhibitors are summarized in Supporting Table S2.

The effect of mutating non-conserved residues in S1, S2 or EL4 in hNET to their hDAT equivalents on the inhibitory potency of the ten inhibitors is summarized in Fig. 4. If non-conserved residues in a specific region are important determinants for inhibitor selectivity, we expected that the potency would be shifted towards the potency at hDAT WT. Transferring the S2 site or EL4 from hDAT into hNET generally induced only modest changes (<3-fold) in the inhibitory potency for the ten inhibitors, demonstrating that non-conserved residues in the S2 site or in the EL4 region of hNET are not important determinants for selective inhibitor recognition. In contrast, transferring the hDAT S1 site into hNET revealed a strikingly well-defined pattern of potency changes for the ten inhibitors. Specifically, the potency of nine of the ten inhibitors was shifted towards their potency at hDAT WT (Fig. 4). The potency of all five NET inhibitors was significantly decreased, whereas the potency was correspondingly increased for the DAT inhibitors bupropion, rimcazole, GBR 12,909 and JHW 007. To examine if the mutational induced changes in inhibitory potency reflect a concomitant change in binding affinity, we determined the affinity of the inhibitors at NET-(DAT S1), NET-(DAT S2) and NET-(DAT EL4) in a [^125^I]β-CIT competition binding assay (Supporting Table S3). Overall, the binding data corroborated our findings from the functional uptake inhibition assay. NET-(DAT S1) induced a significant shift in the binding affinity for five of the ten inhibitors towards hDAT WT affinity, whereas NET-(DAT S2) and NET-(DAT EL4) induced only modest (<3-fold) changes in the binding affinity of the ten inhibitors (Supporting Table S3). Hence, except for bupropion and GBR 12,909, for which NET-(DAT S1) induced an unexpected loss of binding affinity (Supporting Table S3), the binding data supported the findings from the functional uptake inhibition assay. Together, these data strongly suggest that non-conserved residues within the high affinity S1 binding site are key determinants for selective inhibitor recognition in hNET, whereas non-conserved residues in the extracellular entrance pathway (i.e. in the S2 site and EL4 region) only play a minor role for inhibitor selectivity.

### Role of non-conserved residues for inhibitor selectivity in hDAT

**Figure 5:**
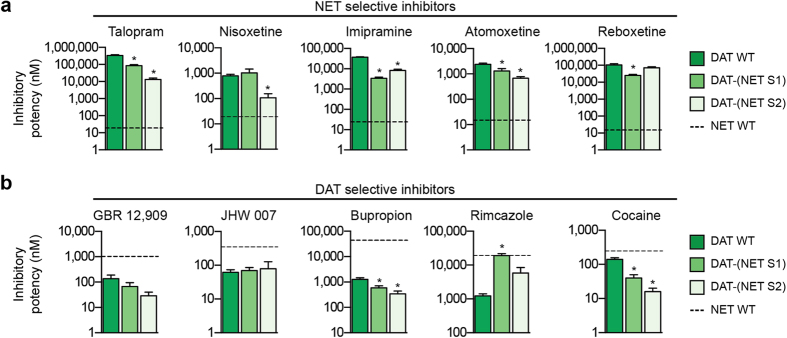
Pharmacological characterization of hDAT mutants. The inhibitory potency of the five NET inhibitors (**a**) and the five DAT inhibitors (**b**) were determined in [^3^H]dopamine uptake inhibition assays at hDAT WT, DAT-(NET S1), DAT-(NET S2) and hNET WT. The bars represent mean ± s.e.m. determined from 3–37 independent experiments each performed in triplicate. The inhibitory potency at hNET WT is indicated by dashed line. Asteriks indicate that the inhibitory potency is significantly different compared to hDAT WT (*p* < 0.05; one-way ANOVA with Dunnett’s multiple comparisons test). The *K*_i_ values for the ten inhibitors are summarized in Supporting Table S2.

Similar to the characterization of inhibitor potency at the hNET mutants, we determined the inhibitory potency of the ten inhibitors at the functionally active hDAT mutants DAT-(NET S1) and DAT-(NET S2) (Fig. 5). As previously mentioned, the DAT-(NET EL4) mutant was devoid of both uptake and binding activity (Fig. 2 and Supporting Table S1), which precluded this mutant from being examined for its pharmacological properties. Surprisingly, the potency of the ten inhibitors was generally much less affected by DAT-(NET S1) and DAT-(NET S2) compared to the effects observed for the corresponding hNET mutants. Specifically, whereas the NET-(DAT S1) mutant induced up to 900-fold change in inhibitor potency, the largest effect induced by the hDAT mutants was a 26-fold gain of talopram potency for DAT-(NET S2) (Fig. 5 and Supplemental Table S2). These results suggest that non-conserved residues in the S1 and S2 sites of hDAT hold a less important role for inhibitor selectivity compared to the equivalent residues, particularly in the S1 site, in hNET. Although the hDAT mutants only affected the inhibitors to a modest extent compared to the hNET mutants, it is noteworthy that DAT-(NET S2) had a larger effect compared to DAT-(NET S1) on five of the inhibitors (talopram, nisoxetine, atomoxetine, bupropion, and cocaine). These findings emphasize that non-conserved S1 residues in hDAT are minor determinants for inhibitor selectivity (Fig. 5). Interestingly, rimcazole stood out as the only compound in which the inhibitory activity was reversed to the level observed in hNET WT by DAT-(NET S1), which suggests that selective inhibitor recognition in hDAT can be regulated by residues in different regions for different inhibitors (Fig. 5). Determination of the binding affinity of the ten inhibitors at DAT-(NET S1) and DAT-(NET S2) using [^125^I]β-CIT competition binding, showed that the mutational induced effects determined in the uptake assay were comparable to the effects determined in the binding assay (Supporting Table S3). Furthermore, we determined the binding affinity of the ten inhibitors at the non-functional DAT-(NET S1S2) mutant that include mutation of all 12 non-conserved residues in the S1/S2 region of hDAT to their hNET equivalents. Generally, the ten inhibitors were not affected more by DAT-(NET S1S2) compared to DAT-(NET S1) or DAT-(NET S2) alone (Supporting Table 3), suggesting that non-conserved residues in the S1 and S2 sites do not act in concert to bestow inhibitor selectivity in hDAT. In summary, characterization of changes in inhibitor potency induced by transferring the hNET S1 and S2 sites into hDAT strongly indicates that non-conserved residues in these regions of hDAT are minor determinants for selective inhibitor recognition, which is in stark contrast to what we found for hNET.

## Discussion

**Figure 6:**
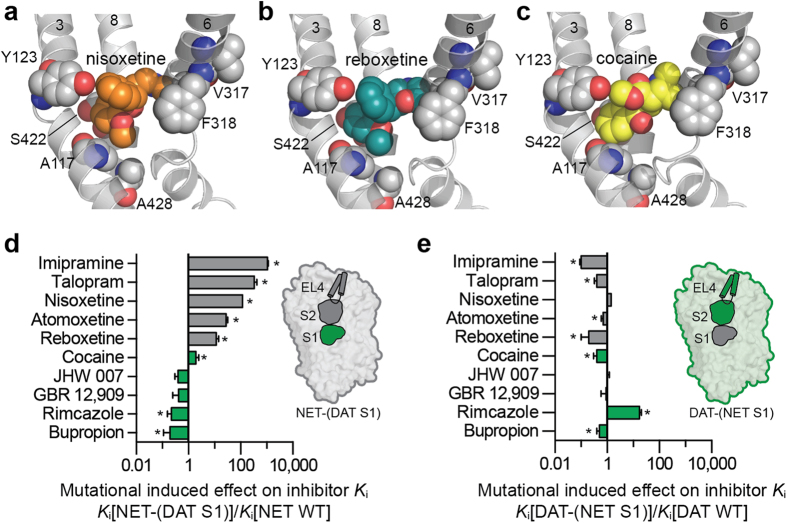
Non-conserved S1 binding site residues are key determinants for inhibitor selectivity in hNET but not in hDAT. The nisoxetine (**a**), reboxetine (**b**) and cocaine (**c**) binding site in dDAT x-ray crystal structures (PDB ID 4XNU, 4XNX, and 4XP4, respectively). The six non-conserved hNET/hDAT residues within the S1 binding site are shown as grey spheres (dDAT numbering). Nisoxetine (**a**), reboxetine (**b**) and cocaine (**c**) are shown as orange, teal and yellow spheres, respectively. (**d**,**e**). Mutational induced effect by the NET-(DAT S1) mutant (**d**) and the DAT-(NET S1) mutant (**e**) on the five NET inhibitors (shown as grey bars) and the five DAT inhibitors (shown as green bars). Asteriks indicate that the mutant induce a significant change in inhibitory potency compared to the WT transporter (*p* < 0.05; one-way ANOVA with Dunnett’s multiple comparisons test). Insets show a graphical representation of the mutants. Inhibitor *K*_i_ values at WT and mutant forms of hNET and hDAT are summarized in Supporting Table S2.

Pharmacological inhibition of the three MATs modulates monoaminergic neurotransmission in the brain and provides foundation for first-line treatments of psychiatric disorders such as depression, anxiety and attention-deficit hyperactivity disorder. The selectivity profile of MAT inhibitors across NET, DAT and SERT is critical for their therapeutic properties. In particular, balancing DAT affinity versus NET and SERT is of special importance due to the potential psychostimulant effect of increasing extracellular dopamine levels through DAT inhibition. However, the molecular basis underlying selective inhibitor recognition in the three MATs remains elusive. In the present study, we took advantage of the high-resolution structural insight into human MATs that has become available via the x-ray crystal structures of dDAT to specifically examine the role of non-conserved residues in the extracellular entrance pathway and within the central S1 binding site for inhibitor selectivity in hNET and hDAT. Specifically, we mutated all non-conserved residues in EL4 and in the S1 and S2 sites to the complementary residues in the other transporter, and thereby principally transferred S1, S2, and EL4 either individually or in combination between hNET and hDAT. Pharmacological characterization of ten representative MAT inhibitors displaying varying degree of selectivity between hNET and hDAT at the resulting mutants revealed that non-conserved residues in S2 and EL4 generally play a minor role for selective inhibitor recognition in both hNET and hDAT (Figs 4 and 5). In contrast, changing the six non-conserved S1 residues in hNET to their hDAT equivalents significantly changed the potency for seven of the ten tested inhibitors towards the level observed at hDAT WT; e.g. providing hNET with a hDAT-like pharmacological profile (Fig. 4). Hence, selective and high affinity inhibitor recognition in hNET seems to be largely controlled by residues in the central S1 binding site. Mapping of the six non-conserved hNET/hDAT residues within the S1 site on inhibitor-bound dDAT x-ray crystal structures shows that these residues are scattered around the S1 site in close proximity of bound inhibitors (Fig. 6). Moreover, in most cases the side chains of these residues are being directly exposed to the pocket interior, hereby contributing to the overall shape of the pocket and potentially provide direct ligand interaction points. This finding corroborates our previous findings, showing that introducing S1 residues from hSERT into hNET transferred high-affinity binding of SERT-selective inhibitors, such as fluoxetine and escitalopram, from hSERT to hNET^29^,^38^. Thus, high-affinity inhibition of hNET is largely determined by the physicochemical nature of the S1 binding site. In comparison, inserting hNET residues into the S1 site of hDAT had a much less pronounced effect on the potency of the same ten inhibitors (Fig. 6). Specifically, compared to the parent WT transporters, NET-(DAT S1) displayed more pronounced shifts in inhibitor *K*_i_ compared to the shifts for the corresponding DAT-(NET S1) mutant for all inhibitors except rimcazole (Fig. 6). The most pronounced differences were observed for talopram and nisoxetine, for which NET-(DAT S1) induced 54- and 128-fold loss of potency, respectively, whereas the equivalent DAT-(NET S1) mutant induced <4-fold change in potency for the two NET inhibitors. Additionally, DAT-(NET S1) induced an unexpectedly increase in the potency of cocaine and bupropion, whereas the mutant did not affect the potency of the DAT inhibitors JHW 007 and GBR 12,909. Together, the mutational data for DAT-(NET S1) show that mutating S1 residues in hDAT to their hNET equivalents do not transfer a NET-like pharmacology to DAT. It should be noted that rimcazole stood out as the only compound for which the DAT-(NET S1) mutant almost fully reversed the selectivity to the level observed at hNET WT (Fig. 5), suggesting that selectivity for some inhibitors are controlled by non-conserved S1 residues in hDAT. However, the overall discrepancy between S1 mutations in hNET and hDAT on the potency of the ten inhibitors examined in the present study suggests that selective and high affinity inhibitor binding is obtained through distinct molecular mechanisms in the two homologous transporters.

The differential pharmacological effects induced by binding site mutants in hNET and hDAT could imply that inhibitors bind to the central S1 site in hNET but to a distinct site in hDAT. However, the recent x-ray crystal structures of dDAT in complex with different inhibitors, including nisoxetine, reboxetine, and cocaine that were employed in the present study, strongly suggest that the high affinity inhibitor binding site is overlapping the central S1 site in hDAT and hNET (Fig. 6)^13^,^17^,^18^. Thus, while the high affinity inhibitor binding site is likely located within the central S1 site in both hNET and hDAT, different molecular determinants seems to regulate inhibitor selectivity in the two transporters. It is noteworthy that the aromatic *ortho*-substituents of nisoxetine and reboxetine that confer high specificity towards hNET over hDAT, bind in a subpocket of the S1 site lined by Ala117 and Ala428 in dDAT (Fig. 6)^17^. Since these residues are not conserved in hNET and hDAT (Ala117/Ala428 in dDAT are equivalent to Ala145/Ala426 in hNET and Ser149/Ser429 in hDAT) this led to the suggestion that specific interactions within this subpocket are key determinants for selectivity towards hNET^17^. This is in accordance with our mutational data for hNET showing that mutation of non-conserved S1 residues in hNET, including Ala145 and Ala426, to their hDAT equivalents, significantly reduced the potency of nisoxetine and reboxetine (up to 168-fold), whereas mutation of non-conserved S1 residues in hDAT, including Ser149 and Ser429, to their hNET equivalents, induced a markedly smaller effect on nisoxetine and reboxetine (up to 4-fold) (Fig. 6). Hence, these data show that inhibitor selectivity cannot simply be explained by differential interactions within a specific subpocket of the high affinity binding site in hDAT, as was proposed based on inhibitor-bound dDAT structures^17^.

Our finding that the pharmacological profile of ten MAT inhibitors at hDAT is largely unaffected by mutation of non-conserved S1 residues, suggest that residues outside the inhibitor binding site are controlling inhibitor selectivity in hDAT. Interestingly, molecular dynamic simulations have revealed that high affinity binding for certain ligands in G protein-coupled receptors is regulated in a distal entry site located at the receptor surface^45^,^46^. Correspondingly, residues in the extracellular entry pathway in hDAT could affect the transient binding steps preceding accommodation of the ligand in the target binding pocket (i.e. the S1 site), and thus comprise a “selectivity filter” for inhibitors. In kinetic terms, the steps preceding ligand binding is reflected in the association rate constant (k_on_) of binding. Recently, differences in k_on_ for some inhibitors, including methylphenidate and desipramine, was shown to be a key determinant for their ability to discriminate between hDAT and hSERT^44^, thus corroborating that molecular determinants outside the high affinity binding site can regulate the selectivity among MATs for some inhibitors.

The location and identity of the determinants outside the high affinity site that control inhibitor affinity in MATs (for example by influencing the association rate of inhibitor binding) remains enigmatic. The extracellular entrance pathway is an obvious candidate, and in the present project we focused on the role of non-conserved residues in the S2 and EL4 regions of the extracellular entry pathway. For S2, we found that non-conserved residues within this region are minor determinants for inhibitor selectivity (Fig. 4). Unfortunately, the DAT-(NET EL4) mutant was devoid of any uptake or binding capacity, which precluded us from examining the role of non-conserved EL4 residues in hDAT. Importantly, as allosteric proteins, MATs are highly dynamic and undergo large-scale conformational changes as part of their function. This implies that all regions within MATs in principle could affect the transporter conformational equilibrium and thereby allosterically affect the region that forms the inhibitor binding site. This has been exemplified in previous studies, showing that disruption of the conformational equilibrium by mutation of residues outside the S1 site affect the binding affinity of DAT inhibitors, including cocaine, GBR 12,909 and bupropion^54^,^55^. Therefore, non-conserved hNET/hDAT residues that are located in regions that regulate the conformational transporter equilibrium could thus be important determinants for inhibitor binding. Further studies are thus warranted to identify the specific differences among hNET and hDAT that affect inhibitors *en route* to their high affinity binding site, including transient transporter-inhibitor interactions in the extracellular entry pathway and conformational transition states, that contribute to MAT inhibitor selectivity.

In summary, our findings demonstrate that non-conserved residues within the primary binding site in hNET and hDAT have differential roles for selective inhibitor recognition, and thus highlight an emerging pattern of a highly complex molecular pharmacology among closely related neurotransmitter transporters. Our data provide new insight into the molecular basis for selectivity of therapeutic and recreational drugs towards hNET and hDAT, which we believe will be important for future design of novel ligands with tailor-made selectivity towards MATs.

## Methods

### Chemicals

[^3^H]dopamine (30–90 Ci/mmol) and [^125^I]β-CIT (2,200 Ci/mmol) was purchased from PerkinElmer (Waltham, MA, USA). Talopram, nisoxetine, imipramine, atomoxetine, GBR 12,909, bupropion and rimzazole were available from the H. Lundbeck A/S compound collection. Reboxetine and cocaine were purchased from Sigma-Aldrich (St. Louis, MO, USA), JHW 007 from Tocris Bioscience (Bristol, UK) and β-CIT from ABX (Radeberg, Germany).

### Molecular biology

As expression vector, a modified version of pCIneo (pCI-IRES-neo) containing the gene encoding either hNET (pCI-IRES-neo-NET) or hDAT (pCI-IRES-neo-DAT) was used. Generation of point mutations in the S1 and S2 sites of hNET and hDAT was performed by site-directed mutagenesis using the QuickChange site-directed mutagenesis kit (Stratagene, Carlsbad, CA, USA). Multiple mutants were generated by introducing one or more mutations into existing mutants by site-directed mutagenesis using the QuickChange mutagenesis kit. The sequence integrity of the mutants was verified by DNA sequencing (GATC Biotech, Cologne, Germany).

For transferring the EL4 region between hNET and hDAT, a unique and silent *Mfe*I restriction site was inserted at Asn338 in hNET and Asn341 in hDAT and a unique and silent *Age*I restriction site was inserted at Thr429 in hNET and Thr432 in hDAT using the QuickChange site-directed mutagenesis kit. Synthetic cDNA encoding the region from Asn338 to Thr429 in hNET [in which the sequence encoding the EL4 region (Leu365 to Trp404) was substituted for the corresponding hDAT sequence] and Asn341 to Thr432 in hDAT [in which the sequence encoding the EL4 region (Leu368 to Trp407) was substituted for the corresponding hNET sequence] was purchased from GeneArt. The synthetic DNA was digested with *Mfe*I and *Age*I and then ligated into *Mfe*I/*Age*I digested pCI-IRES-neo-NET and pCI-IRES-neo-DAT using the Rapid DNA ligation kit (Roche, Mannheim, Germany). The sequence integrity was verified by restriction digests and DNA sequencing (GATC Biotech, Cologne, Germany).

### Cell culturing and expression of hNET and hDAT

COS-7 cells were cultured in Dulbecco’s Modified Eagle Medium (DMEM) supplemented with 10% foetal bovine serum, 100 U/mL penicillin, and 100 μg/mL streptomycin at 37 °C in a humidified 5% CO_2_ environment. Cells were transfected using TransIT LT-1 DNA transfection reagent by following the procedure supplied by the manufacturer, and dispensed into either poly-d-lysine coated white 96-well plates (for uptake assays) or 150 mm tissue culture Petri plates (for membrane preparations).

### [^3^H]dopamine uptake assays

Uptake assays were performed 40–48 h after transfection. Transiently transfected COS-7 cells growing in 96-well plates were washed twice with PBSCM buffer (137 mM NaCl, 2.7 mM KCl, 4.3 mM Na_2_HPO_4_, 1.4 mM KH_2_PO_4_, 0.1 mM CaCl_2_ and 0.5 mM MgCl_2_) on an ELx50 automated microplate strip washer (BioTek Instruments, Winooski, VT, USA). In all uptake experiments, the PBSCM buffer was supplemented with 1 mM ascorbic acid and 1 μM of the catechol-*O*-methyltransferase inhibitor Ro 41–0960 (Sigma-Aldrich) and pH was adjusted to 7.4.

In inhibition studies (IC_50_ determinations), cells were preincubated with 40 μL of PBSCM per well with no or increasing concentrations of inhibitor for 30 min before addition of 10 μL of PBSCM containing [^3^H]dopamine (final concentration of [^3^H]dopamine: 25–50 nM). Uptake was allowed to proceed for 5 min at room temperature, before uptake was terminated by washing three times with PBSCM. For determination of *K*_M_ values, cells were incubated with increasing concentrations of unlabelled dopamine in PBSCM and 25–50 nM [^3^H]dopamine at room temperature for 5 min. Uptake was terminated as described above. The amount of accumulated [^3^H]dopamine was determined by solubilising cells in scintillant cocktail (MicroScint-20) followed by counting of plates in a Packard TopCounter. Non-specific uptake was determined as uptake in non-transfected cells and total uptake was determined in the presence of PBSCM buffer alone. Assays were carried out in triplicate and repeated at least three times.

### Cell membrane preparation and [^125^I]β-CIT binding assay

Cell membranes were prepared 40–48 h after transfection. Transiently transfected COS-7 cells growing in 150 mm tissue culture Petri plates were washed with PBS supplemented with 1 mM EDTA to detach from the plate. Cell suspension was centrifuged (700 × *g*) for 5 min, and the cell pellet was suspended in cold H_2_O and frozen at −20 °C for at least 1 h. The suspension was thawed on ice and subjected to 10–15 passages through a 21 gauge needle to disrupt cells. Homogenate was transferred to 2 mL tubes and centrifuged at 18.000 × *g* for 30 min at 4 °C. Supernatant was aspirated, and the pellet was suspended in PBSCM buffer. Protein concentration was determined by the BCA method using Pierce BCA Protein Assay. Membranes were used directly for binding assays or stored at −80 °C until use.

For saturation binding studies, increasing concentrations of [^125^I]β-CIT diluted 1:20 with unlabelled β-CIT and 2–30 μg total membrane protein was combined in 96-well plates and total volume per well was adjusted to 150 μL with PBSCM. Binding was allowed to equilibrate for 2 h on ice. Subsequently, membranes were transferred to 96-well glass fiber filter plates (Unifilter C; PerkinElmer) preincubated with 0.1% polyethyleneimine (30 μL per well) by using a Packard Bell cell harvester and washed four times with water. Nonspecific binding was determined in parallel at membranes from nontransfected COS-7 cells. Filter plates were dried and soaked in scintillant cocktail (MicroScint-0) followed by counting of plates in a Packard TopCounter. For competition binding assays, 2–30 μg total membrane protein was incubated with 0.2 nM [^125^I]β-CIT in the presence of no or increasing concentration of inhibitor in PBSCM by using the same protocol as for saturation binding experiments. Binding assays were carried out in duplicate and repeated at least three times.

### Data analysis

All data analysis was performed using GraphPad Prism version 6.0 (GraphPad Software, La Jolla, CA, USA). The inhibitory potency (*K*_i_) for inhibitors were calculated from IC_50_ values determined in functional uptake assays by using the equation: *K*_i_ = IC_50_/(1 + ([L]/*K*_M_)), where [L] is the concentration of [^3^H]dopamine and *K*_M_ is the Michaelis-Menten constant for dopamine. The binding affinity (*K*_i_) for inhibitors were calculated from IC_50_ values determined in competition binding assays by using the equation: *K*_i_ = IC_50_/(1 + ([L]/*K*_D_)), where [L] is the concentration of [^125^I]β-CIT and *K*_D_ is the dissociation constant for β-CIT. All data are expressed as mean ± s.e.m. from at least three independent experiments carried out in triplicate (for uptake assays) or duplicate (for binding assays). Statistical analyses were performed by using one-way analysis of variance (ANOVA). Values of *p* < 0.05 were considered statistically significant.

## Additional Information

**How to cite this article**: Andersen, J. *et al.* Binding site residues control inhibitor selectivity in the human norepinephrine transporter but not in the human dopamine transporter. *Sci. Rep.* **5**, 15650; doi: 10.1038/srep15650 (2015).

## Supplementary Material

Supporting Information
