# Supplementary material for: Binding site residues control inhibitor selectivity in the human norepinephrine transporter but not in the human dopamine transporter
Source: Sci Rep. 2015 Oct 27;5:15650. doi: 10.1038/srep15650 (PMC4621520; doi:10.1038/srep15650)
Supplement: Supporting Information [file srep15650-s1.pdf]

Supporting Material

Binding site residues control inhibitor selectivity in the human norepinephrine transporter but not in the human dopamine transporter

Jacob Andersen[1,*], Kristoffer B. Ringsted[1], Benny Bang-Andersen[1,2], Kristian Strømgaard[1], and Anders S. Kristensen[1]

[1]Department of Drug Design and Pharmacology, University of Copenhagen, Jagtvej 162, DK-2100 Copenhagen, Denmark, [2]Lundbeck Research Denmark, H. Lundbeck A/S, Ottiliavej 9, DK-2500 Valby, Denmark.

Correspondence and requests for materials should be addressed to:
Jacob Andersen, Department of Drug Design and Pharmacology, University of Copenhagen, Jagtvej 162, DK-2100 Copenhagen, Denmark. E-mail: jaa@sund.ku.dk. Tel.: +45 3533 6388. Fax: +45 3533 6040.

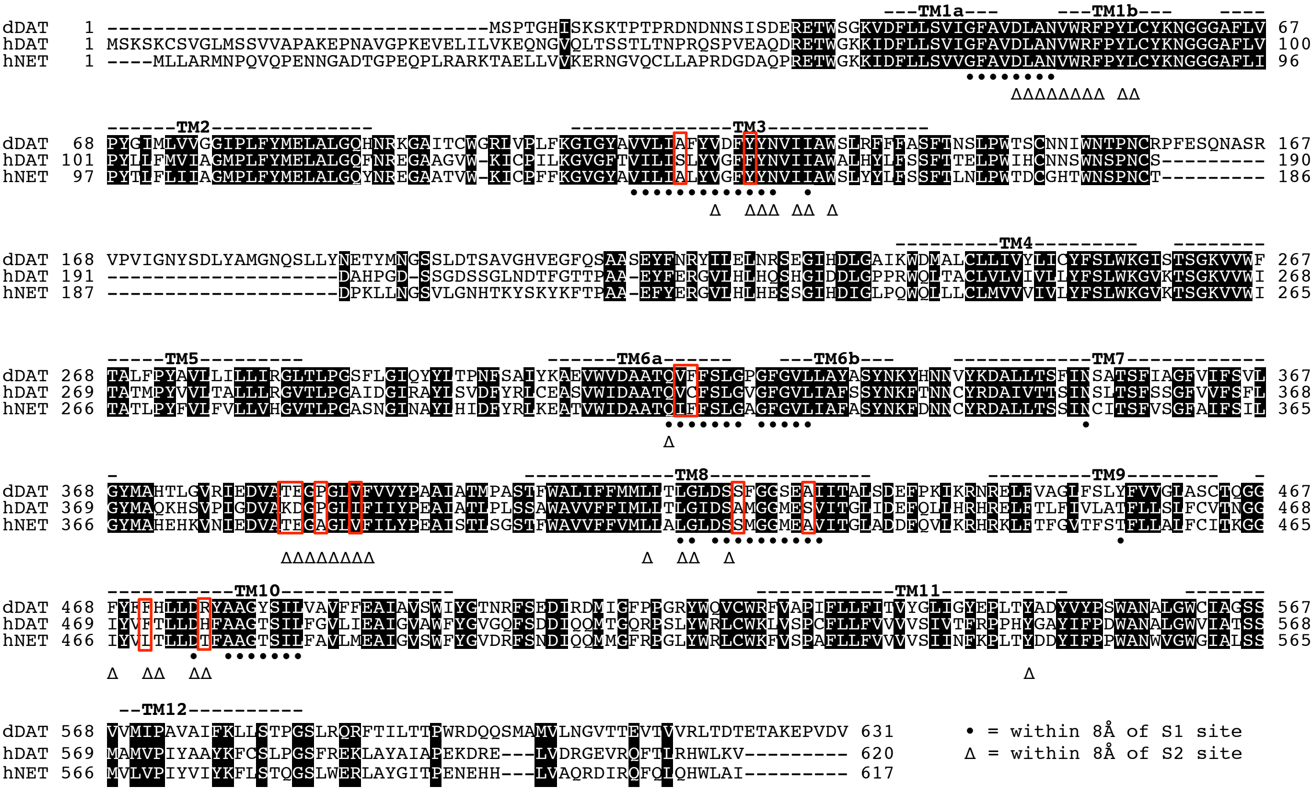

Supporting Figure S1: Amino acid sequence alignment of dDAT, hDAT and hNET.
The regions encompassing the 12 transmembrane spanning helices in the dDAT X-ray crystal structure are indicated above the sequence alignment. Conserved and similar residues are shown as white text on black background. The S1 site was defined as the nortriptyline binding site in dDAT (PDB ID 4M48). Residues within 8Å of the S1 site is indicated with black circles. The S2 site was defined as the equivalent site in dDAT as the imipramine binding site in LeuT (PDB ID 2Q72), and residues within 8Å of the S2 site is indicated with open triangles. Non-conserved hNET/hDAT residues within the S1 and S2 sites are highlighted with red squares. According to these definitions, 6 out of 56 residues (11%) within 8Å of the S1 site are not conserved between hNET and hDAT, while 7 out of 36 residues (19%) within 8Å of the S2 site are not conserved between hNET and hDAT.

Mutant[a]	[^{3}H]dopamine uptake		[^{125}I]β-CIT binding
	K_M (μM)	V_{max} (% of WT)	K_d (nM)
hNET WT	0.35 ± 0.04	100	16 ± 3.2
NET-(DAT S1)	0.12 ± 0.02	44 ± 8.0	1.4 ± 0.2
NET-(DAT S2)	0.07 ± 0.01	41 ± 3.5	21 ± 1.5
NET-(DAT S1S2)	0.23 ± 0.05	84 ± 4.8	23 ± 7.9
NET-(DAT EL4)	0.26 ± 0.04	81 ± 11	16 ± 1.8
NET-(DAT S1S2 EL4)	N.F.	N.F.	N.B.
hDAT WT	1.6 ± 0.9	100	1.0 ± 0.2
DAT-(NET S1)	0.75 ± 0.06	73 ± 9.0	0.68 ± 0.11
DAT-(NET S2)	0.52 ± 0.05	33 ± 6.0	0.46 ± 0.13
DAT-(NET S1S2)	N.F.	N.F.	0.12 ± 0.04
DAT-(NET EL4)	N.F.	N.F.	N.B.
DAT-(NET S1S2 EL4)	N.F.	N.F.	N.B.

Supporting Table S1: Effect of hNET and hDAT mutants on [^{3}H]dopamine uptake and [^{125}I]β-CIT binding properties.
K_M and V_{max} values for dopamine was determined from functional [^{3}H]dopamine uptake competition assays using COS-7 cells expressing WT or mutant forms of hNET or hDAT as described in *Methods*. The data from [^{3}H]dopamine uptake competition assays were transformed to saturation curves (as shown on Figure 2) that were used for calculation of V_{max} values. Data from the [^{3}H]dopamine uptake assays represent mean ± s.e.m. from 4 – 22 independent experiments each performed in triplicate. K_d values for β-CIT was determined from [^{125}I]β-CIT saturation binding assays using COS-7 membranes expressing WT or mutant forms of hNET or hDAT as described in *Methods.* Data from the [^{125}I]β-CIT saturation binding assays represent mean ± s.e.m. from 4 – 15 independent experiments each performed in duplicate. N.F. = non-functional. N.B. = no binding capacity.
[a]The hNET and hDAT mutants include the following point-mutations:

NET-(DAT S1): hNET-A145S-Y151F-I315V-F316C-S420A-A426S

NET-(DAT S2): hNET-Y151F-T381K-E382D-A384P-V387I-L469F-T474H

NET-(DAT S1S2): hNET-A145S-Y151F-I315V-F316C-T381K-E382D-A384P-V387I-S420A-A426S-L469F-T474H

NET-(DAT EL4): hNET-H370Q-E371K-K373S-N375P-E377G-T381K-E382D-A384P-V387I-L390I-S396A-S399P-G400L-T402S-F403A

NET-(DAT S1S2 EL4): hNET-A145S-Y151F-I315V-F316C-H370Q-E371K-K373S-N375P-E377G-T381K-E382D-A384P-V387I-L390I-S396A-S399P-G400L-T402S-F403A-S420A-A426S-L469F-T474H

DAT-(NET S1): hDAT-S149A-F155Y-V318I-C319F-A423S-S429A

DAT-(NET S2): hDAT-F155Y-K384T-D385E-P387A-I390V-F472L-H477T

DAT-(NET S1S2): hDAT-S149A-F155Y-V318I-C319F-K384T-D385E-P387A-I390V-A423S-S429A-F472L-H477T

DAT-(NET EL4): hDAT-Q373H-K374E-S376K-P378N-G380E-K384T-D385E-P387A-I390V-I393L-A399S-P402S-L403G-S405T-A406F

DAT-(NET S1S2 EL4): hDAT-S149A-F155Y-V318I-C319F-Q373H-K374E-S376K-P378N-G380E-K384T-D385E-P387A-I390V-I393L-A399S-P402S-L403G-S405T-A406F-A423S-S429A-F472L-H477T

	hNET WT	n	NET-(DAT S1)	n	NET-(DAT S2)	n	NET-(DAT EL4)	n
	K_i (nM)	n	K_i (nM)	n	K_i (nM)	n	K_i (nM)	n
Talopram	19 ± 3	18	3,933 ± 611*	11	37 ± 10	12	61 ± 18	6
Nisoxetine	1.9 ± 0.3	8	320 ± 83*	5	1.8 ± 0.3	5	12 ± 6	3
Imipramine	24 ± 4	7	21,993 ± 2,385*	3	94 ± 40	4	188 ± 74	3
Atomoxetine	15 ± 2	9	418 ± 104*	5	22 ± 6	5	11 ± 6	3
Reboxetine	15 ± 2	12	154 ± 32*	6	34 ± 7	7	11 ± 3	3
GBR 12,909	1,024 ± 259	6	418 ± 166	4	617 ± 281	4	1,706 ± 794	3
JHW007	349 ± 90	6	185 ± 44	5	685 ± 244	4	276 ± 29	3
Bupropion	44,051 ± 11,188	8	3,807 ± 1,117*	6	52,757 ± 11,330	7	6,268 ± 2,208	3
Rimcazole	19,122 ± 2,742	6	3,295 ± 843*	4	11,055 ± 2,350	4	40,667 ± 10,568	3
Cocaine	246 ± 45	8	445 ± 140	5	1,683 ± 463*	6	468 ± 93	3

	hDAT WT	n	DAT-(NET S1)	n	DAT-(NET S2)	n
	K_i (nM)	n	K_i (nM)	n	K_i (nM)	n
Talopram	333,104 ± 32,164	37	84,088 ± 13,054*	8	12,912 ± 2,736*	9
Nisoxetine	782 ± 132	6	1,024 ± 441	3	108 ± 46*	3
Imipramine	37,322 ± 1,891	6	3,359 ± 554*	3	8,220 ± 1,226*	3
Atomoxetine	2,395 ± 279	12	1,316 ± 307*	5	679 ± 108*	5
Reboxetine	104,288 ± 20,350	9	25,088 ± 4,117*	5	71,652 ± 10,490	3
GBR 12,909	136 ± 52	6	67 ± 28	3	29 ± 11	3
JHW007	62 ± 12	8	70 ± 16	5	78 ± 49	5
Bupropion	1,236 ± 210	14	578 ± 112*	10	342 ± 98*	7
Rimcazole	1,229 ± 176	7	18,872 ± 3,006*	4	5,845 ± 2,662	3
Cocaine	140 ± 17	8	40 ± 10*	4	16 ± 4*	4

Supporting Table S2: Inhibitory potency of the ten inhibitors at hNET (*upper*) and hDAT (*lower*) mutants. The inhibitory potency (K_i) of the ten inhibitors was determined from [^{3}H]dopamine competition uptake assays using COS-7 cells expressing WT or mutant forms of hNET or hDAT as described in *Methods.* Data represent mean ± s.e.m. from 3 – 37 independent experiments each performed in triplicate. Asteriks indicate significantly different K_i value compared to WT transporter (p < 0.05; one-way ANOVA with Dunnett's multiple comparisons test).

	hNET WT K_i (nM)	n	NET-(DAT S1) K_i (nM)	n	NET-(DAT S2) K_i (nM)	n	NET-(DAT EL4) K_i (nM)	n
Talopram	19 ± 6	10	151 ± 34*	4	17 ± 6	4	18 ± 2	3
Nisoxetine	5.8 ± 3.5	12	169 ± 56*	5	2.7 ± 1.3	3	4.3 ± 0.7	3
Imipramine	12 ± 4	7	1.1 ± 0.2	6	30 ± 2.9	3	46 ± 22*	4
Atomoxetine	1.6 ± 0.6	7	4.4 ± 0.8*	3	1.3 ± 0.2	3	3.8 ± 1.1*	3
Reboxetine	10 ± 1	6	49 ± 3*	3	21 ± 3.9	3	29 ± 8*	3
GBR 12,909	391 ± 91	5	3,065 ± 438*	3	140 ± 22	3	471 ± 86	3
JHW007	860 ± 111.4	7	1,288 ± 782	4	1,208 ± 625	4	337 ± 99	6
Bupropion	10,098 ± 2,268	8	60,616 ± 18,796*	5	10,471 ± 2,267	4	10,338 ± 2,271	3
Rimcazole	14,801 ± 2,381	6	543 ± 74*	3	10,105 ± 829	3	12,614 ± 1,779	3
Cocaine	925 ± 302	7	500 ± 192	3	1,631 ± 633	4	1,040 ± 163	3

	hDAT WT K_i (nM)	n	DAT-(NET S1) K_i (nM)	n	DAT-(NET S2) K_i (nM)	n	DAT-(NET S1S2) K_i (nM)	n
Talopram	137,029 ± 35,130	5	105,871 ± 15,904	6	70,064 ± 16,528	6	26,511 ± 4,190*	6
Nisoxetine	1,513 ± 491	5	3,311 ± 662	5	768 ± 134*	5	642 ± 169	5
Imipramine	23,904 ± 5,808	4	9,087 ± 2,566*	5	15,373 ± 2,256	5	8,979 ± 1,659*	5
Atomoxetine	3,394 ± 284	3	1,167 ± 55*	3	496 ± 72*	3	327 ± 52*	3
Reboxetine	36,698 ± 4,567	3	36,966 ± 3,312	3	60,712 ± 4,862*	3	37,223 ± 7,572	3
GBR 12,909	57 ± 14	3	89 ± 15	4	26 ± 3	4	17 ± 6	4
JHW007	49 ± 7	5	61 ± 10	5	34 ± 2	5	70 ± 8	5
Bupropion	810 ± 207	3	520 ± 48	3	367 ± 21	3	416 ± 94	3
Rimcazole	745 ± 195	3	8,412 ± 427*	3	7,467 ± 320*	3	7,568 ± 77*	3
Cocaine	372 ± 22	3	76 ± 10*	3	51 ± 7*	3	49 ± 8*	3

Supporting Table S3: Binding affinity of the ten inhibitors at hNET (*upper*) and hDAT (*lower*) mutants. The binding affinity (K_i) of the ten inhibitors was determined from [^{125}I]β-CIT competition binding assays using COS-7 membranes expressing WT or mutant forms of hNET or hDAT as described in *Methods.* Data represent mean ± s.e.m. from 3 – 12 independent experiments each performed in dublicate. Asteriks indicate significantly different K_i value compared to WT transporter ($p < 0.05$; one-way ANOVA with Dunnett's multiple comparisons test).
